# Distinguishing Progressive Multifocal Leukoencephalopathy From Cerebral Toxoplasmosis in HIV: A Case Report

**DOI:** 10.7759/cureus.90349

**Published:** 2025-08-17

**Authors:** Htar Htet Htet Wai, Su M Aung, Phyu Hnin Ei, Su Sandar, Robert Wekesa

**Affiliations:** 1 Acute Medicine, Lincoln County Hospital, Lincoln, GBR; 2 Acute Medicine, United Lincolnshire Hospitals NHS Trust, Lincoln, GBR; 3 Respiratory Medicine, United Lincolnshire Teaching Hospitals NHS Trust, Lincoln, GBR; 4 Pediatrics, Glangwili Hospital, Carmarthen, GBR

**Keywords:** hiv, jc virus pcr, progressive multifocal leucoencephalopathy, ring-enhancing lesions, toxoplasmosis

## Abstract

Progressive multifocal leukoencephalopathy and cerebral toxoplasmosis are serious opportunistic infections in patients with advanced HIV infection. Diagnosis is often challenging due to overlapping clinical and radiological features. We present the case of a 40-year-old woman with a three-week history of expressive dysphasia, headache, and right arm weakness. Imaging revealed multiple enhancing brain lesions, initially raising concern for metastatic cancer. An extensive malignancy workup was unrevealing. Lumbar puncture confirmed JC virus in the CSF, and further testing identified previously undiagnosed HIV with a CD4 count of 39 cells/μL, positive Toxoplasma IgG, and hepatitis C coinfection. Treatment with antiretroviral therapy, co-trimoxazole, and corticosteroids was initiated. The patient subsequently developed suspected immune reconstitution inflammatory syndrome and new-onset seizures.

## Introduction

Progressive multifocal leukoencephalopathy (PML) and cerebral toxoplasmosis are severe opportunistic infections of the CNS, most commonly observed in individuals with advanced HIV/AIDS, particularly when CD4+ T-cell counts fall below 200 cells/μL [1]. Both conditions can present with overlapping neurological symptoms, including focal deficits, seizures, altered mental status, and headaches [2]. MRI often reveals ring-enhancing lesions in both diseases, which can complicate the differential diagnosis [3]. Delays in accurate identification may lead to rapid clinical deterioration and increased mortality.

PML is caused by reactivation of the JC virus and is typically characterized by non-enhancing demyelinating lesions without significant swelling or mass effect [4]. By contrast, cerebral toxoplasmosis, caused by the parasite *Toxoplasma gondii*, often presents with multiple lesions accompanied by edema and mass effect, most commonly involving the basal ganglia and corticomedullary junction [5]. In the absence of a known HIV diagnosis, such imaging findings may mimic brain metastases or primary brain tumors, leading to diagnostic delays.

We report a challenging case of a patient with previously undiagnosed HIV who presented with neurological symptoms and brain imaging suggestive of metastatic disease. Further evaluation confirmed coinfection with the JC virus and *Toxoplasma*, underscoring the importance of considering opportunistic infections in patients with unexplained neurological findings.

## Case presentation

History

A 40-year-old woman presented to the emergency department with a three-week history of headaches, expressive aphasia, and progressive weakness in her right upper limb. Her vital signs were stable on admission (temperature: 36.8°C, heart rate: 82 bpm, blood pressure: 118/74 mmHg, respiratory rate: 16/min, and oxygen saturation: 98% on room air). Neurological examination revealed 3/5 motor strength in the right upper limb. Examination of other systems was unremarkable.

Initial investigations

**Figure 1:**
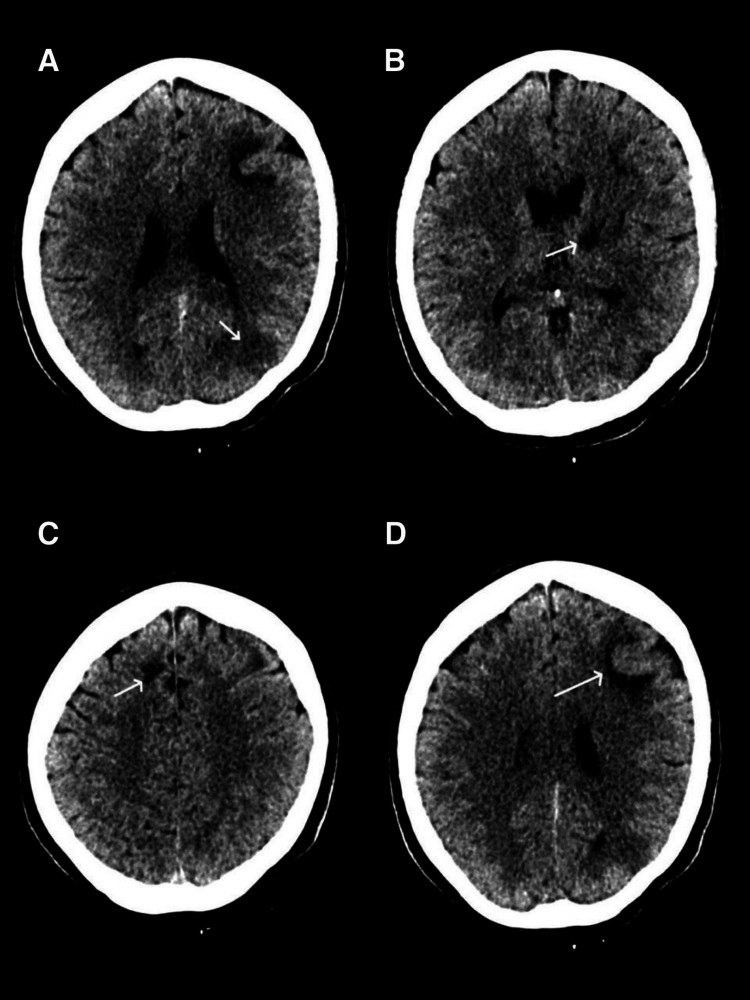
Non-contrast CT of the brain (A) Hypodense area in the left parietal lobe (white arrow), consistent with an acute infarct. (B) Chronic hypodensity in the left ganglio-capsular region (white arrow). (C, D) Irregular hypodensities in the bilateral frontal lobes (white arrows).

**Figure 2:**
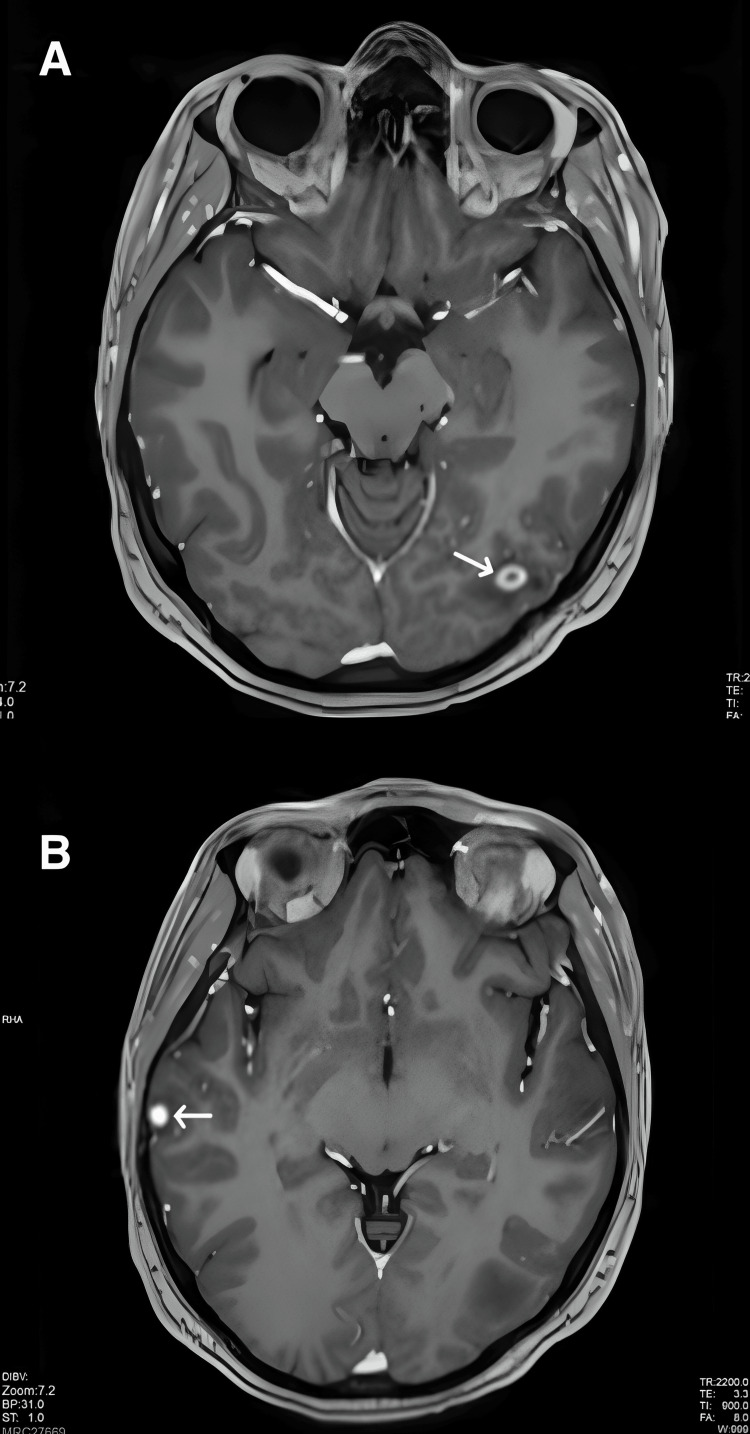
Contrast-enhanced MRI of the brain (A) Enhancing lesion in the left occipital lobe (white arrow). (B) Enhancing lesion in the right temporal lobe (white arrow).

Initial investigations showed normal results for her full blood count, urea and electrolytes, liver function tests, and C-reactive protein. A chest X-ray demonstrated no acute pathology, and an ECG showed normal sinus rhythm. A non-contrast CT scan of the head demonstrated a focal hypodense area in the left parietal lobe, suggestive of an acute infarct. Additionally, irregular hypodensities were noted in both frontal lobes and the left gangliocapsular region, consistent with chronic infarcts (Figure 1). A subsequent MRI of the brain revealed contrast-enhancing lesions in the left occipital lobe and right temporal lobe (Figure 2). These findings raised the suspicion of metastatic brain lesions.

Workup and management

During the initial workup, extensive investigations were conducted to exclude malignancy. These included a CT scan of the chest, abdomen, and pelvis, a PET-CT scan, mammography, and a bone marrow biopsy, all of which were negative. The patient was started on corticosteroids by the neurology team, resulting in notable symptomatic improvement. She was discharged on a weaning dose of steroids after three weeks in the hospital while awaiting further results.

One week after discharge, the patient was readmitted due to worsening weakness in her right upper limb. A lumbar puncture revealed elevated CSF protein levels of 466 mg/L and a white cell count of 1 cell/µL. CSF PCR testing confirmed the presence of JC virus DNA. Other viral studies in CSF were normal (Table 1). Subsequent investigations revealed a positive HIV test and positive serum* T. gondii *IgG (Table 2). Serial hematological parameters, including hemoglobin, white cell count with differential, and platelet counts, are summarized in Table 3 to highlight trends during hospital admissions and after initiation of antiretroviral therapy (ART).

**Table 1:** CSF findings This table summarizes the CSF analysis obtained during the patient’s diagnostic workup. JC virus was detected by PCR, with a viral load of 2,630 IU/mL, confirming the diagnosis of PML. Protein levels were elevated, while all other viral and bacterial PCR assays were negative. CMV, cytomegalovirus; EBV, Epstein-Barr virus; PML, progressive multifocal leukoencephalopathy

Test	Result
CSF protein	466 mg/L (reference: 150-400 mg/L)
WBC	1 cell/µL (reference: <1 cell/µL)
Culture	No growth
Cryptococcal antigen	Negative
JC virus PCR	Detected
JC virus viral load	2,630 IU/mL
Treponemal PCR	Not detected
Adenovirus PCR	Not detected
CMV PCR	Not detected
EBV PCR	Not detected
Enterovirus PCR	Not detected

**Table 2:** Summary of blood test results This table presents the patient’s relevant serological and immunological findings, supporting the diagnosis of HIV, prior exposure to *Toxoplasma gondii*, and coinfections. CD4, cluster of differentiation 4; TB, tuberculosis; VDRL, Venereal Disease Research Laboratory (test for syphilis)

Blood test	Result
HIV antibody	Positive
Hepatitis C genotype 3a	Detected
Toxoplasma IgG	Positive
Toxoplasma IgM	Negative
CD4 cell count	39 cells/uL
Quantiferon TB	Negative
VDRL	Positive

**Table 3:** Serial FBC results during hospital admissions compared with normal values The patient’s FBC demonstrated relatively stable hemoglobin levels throughout both admissions, remaining within normal limits. The WCC and differential counts (neutrophils and lymphocytes) were also within normal ranges, with only minor fluctuations. Platelet counts were slightly below normal at the first admission (124 × 10⁹/L) but improved following ART initiation and remained closer to normal preterminally (150 × 10⁹/L). Overall, apart from mild initial thrombocytopenia, the patient’s systemic hematologic profile remained largely unremarkable during her hospital course, with no significant cytopenias prior to death. ART, antiretroviral therapy; FBC, full blood count; Hb, hemoglobin; WCC, white cell count

Parameter	First admission	Second admission (initial)	Two weeks after ART initiation	Preterminal (before death)	Normal values
Hb (g/L)	137	132	141	131	120-160
WCC (×10⁹/L)	6.3	5.8	6.4	4.6	4-11
Platelets (×10⁹/L)	124	141	168	150	150-400
Neutrophils (×10⁹/L)	4.55	4.16	4.1	3.9	2-7
Lymphocytes (×10⁹/L)	1.22	1.13	1.38	1.2	1-4

**Figure 3:**
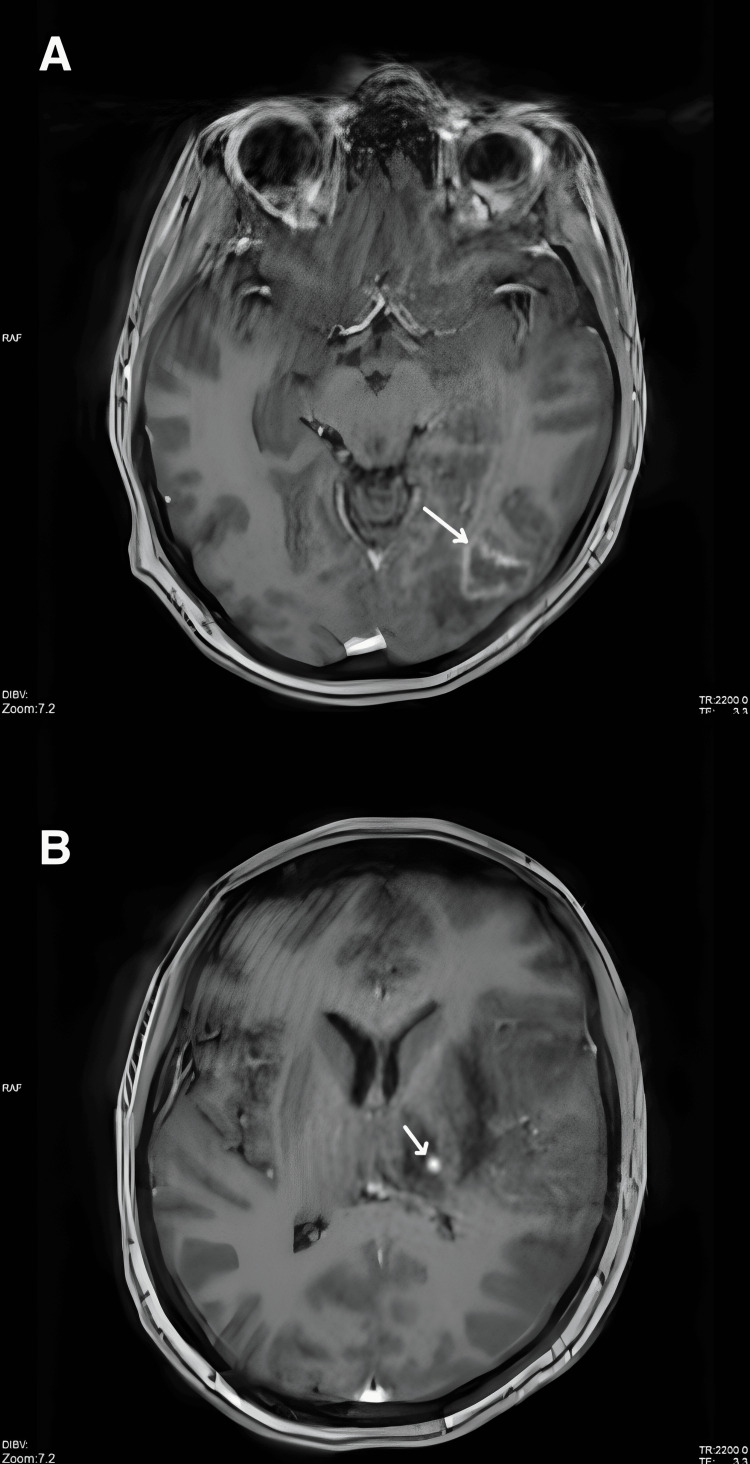
Repeat MRI of the brain (A) Progression of the contrast-enhancing lesion in the left occipital lobe (white arrow). (B) Newly developed contrast-enhancing nodule in the left thalamus (white arrow), suggestive of disease progression.

A repeat MRI demonstrated progression of the left occipital lesion along with new contrast-enhancing nodules (Figure 3).

**Figure 4:**
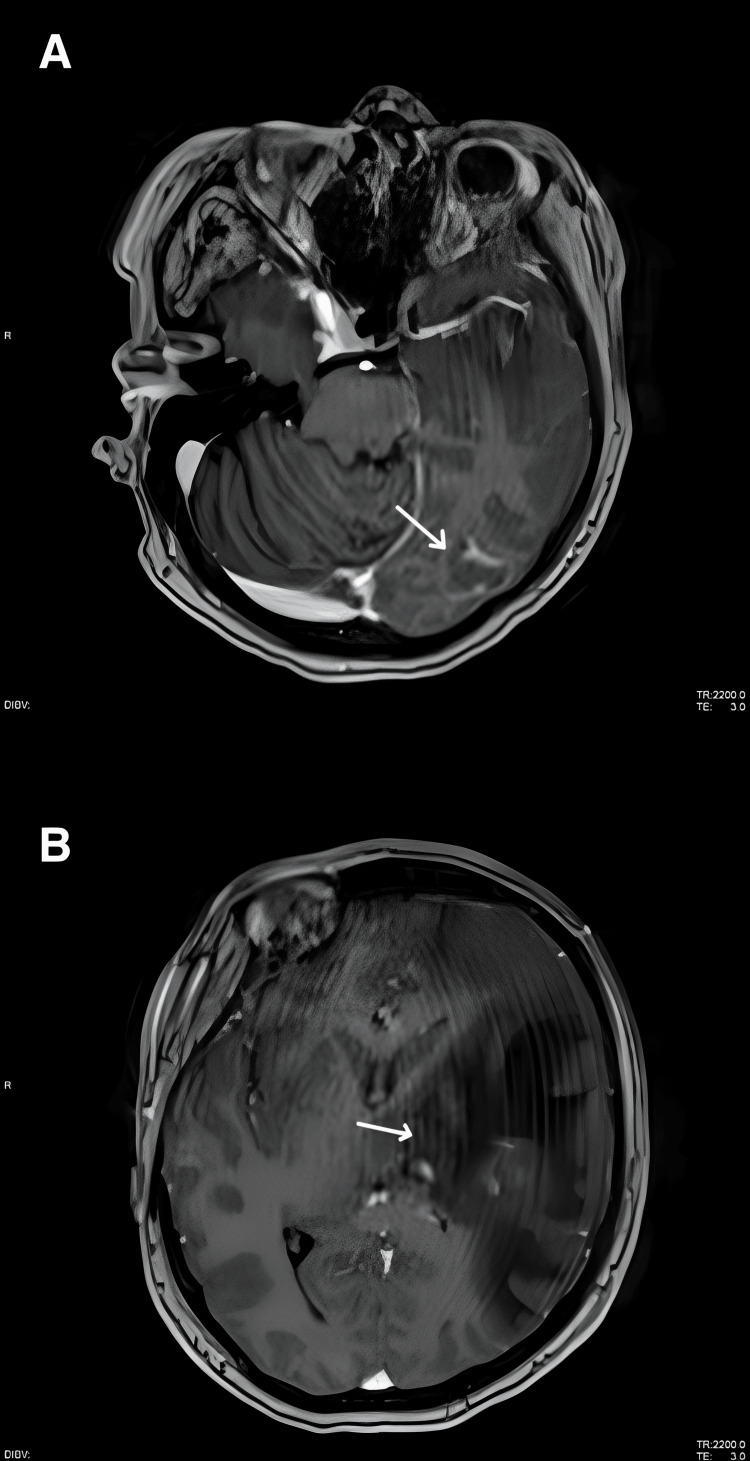
Follow-up contrast-enhanced MRI performed two weeks after treatment initiation (A) Persistent enhancement of the left occipital lesion with minimal interval improvement (white arrow). (B) Increased enhancement of the left thalamic lesion (white arrow), suggestive of progressive disease.

The detection of JC virus DNA in the CSF, together with a new diagnosis of HIV infection and profound immunosuppression (CD4 count 39 cells/μL), shifted the diagnostic focus toward opportunistic CNS infections. The patient was commenced on ART (Truvada: tenofovir DF 300 mg + emtricitabine 200 mg, and dolutegravir 50 mg), along with co-trimoxazole 480 mg for both prophylaxis and treatment of toxoplasmosis. Two weeks after the initiation of treatment, a repeat MRI scan showed only minimal improvement in the previously affected occipital lesion, while the left thalamus demonstrated increased enhancement (Figure 4). At the same time, progression of PML-related demyelinating changes spread diffusely throughout both cerebral hemispheres, worsening the cerebral edema and mass effect.

Given the initial profound immunosuppression and the subsequent clinical and radiologic deterioration, PML-IRIS was suspected. Empirical corticosteroid therapy was initiated. One week later, the patient developed status epilepticus and was transferred to the intensive care unit. Unfortunately, despite aggressive treatment, she passed away due to complications from aspiration pneumonia and progression of PML.

## Discussion

We report a challenging case of a patient with previously undiagnosed HIV who presented with neurological symptoms and brain imaging findings initially suggestive of metastatic disease. Further evaluation confirmed coinfection with the JC virus and *T. gondii*, underscoring the importance of considering opportunistic infections in patients with unexplained neurological findings.

PML is a demyelinating disease caused by reactivation of the JC virus in immunocompromised hosts. It typically presents subacutely with focal neurological deficits such as weakness and aphasia, as observed in our patient [6,7]. Diagnosis is established through MRI findings of multifocal white matter lesions and detection of JC virus DNA in CSF by PCR. The cornerstone of treatment is immune restoration with ART, though this can be complicated by immune reconstitution inflammatory syndrome (IRIS). IRIS occurs when ART restores pathogen-specific immune responses in severely immunocompromised patients, leading to an exaggerated inflammatory reaction against residual antigens or viable pathogens. In PML, IRIS arises from a rapid increase in CD4+ T cells and activation of JC virus-specific immune responses, which trigger intense inflammation within the CNS. This immune-mediated process can exacerbate neurological symptoms and radiological findings, such as contrast enhancement and edema surrounding lesions, despite virological control of the JC virus. Although the inflammatory response reflects immune recovery, it may also cause tissue damage and clinical deterioration, necessitating corticosteroid therapy to modulate the immune response [8,9].

Cerebral toxoplasmosis is the most common opportunistic CNS infection in patients with HIV, particularly when CD4 counts fall below 100 cells/μL. It often presents with headaches, focal neurological signs, and ring-enhancing lesions on MRI, consistent with findings in our case [10]. Diagnosis is supported by positive *T. gondii* IgG serology. Treatment typically involves pyrimethamine-based regimens or high-dose co-trimoxazole, which also provides prophylaxis against toxoplasmosis in immunocompromised individuals [11].

Our patient presented late in the course of HIV infection with profound immunosuppression (CD4 count 39 cells/μL), highlighting the persistent challenge of late HIV diagnosis in Europe. Routine HIV screening is recommended by both the European Centre for Disease Prevention and Control and the World Health Organization, particularly in high-risk populations and healthcare settings, to facilitate early detection and treatment [12,13]. While the UK is considered a low-prevalence country for HIV overall, certain urban areas, such as London, Manchester, and Brighton, have higher rates of diagnosed infections (≥2 cases per 1,000 population aged 15-59 years), meeting the threshold for high-prevalence settings. In these areas, as well as among high-risk populations, routine opt-out HIV testing is recommended to improve early diagnosis and reduce late presentations [14,15].

Seizures are recognized complications of both toxoplasmosis and PML, particularly during IRIS, likely due to cortical irritation and increased inflammation. Antiepileptic drugs are critical for seizure management, although specific guidelines for seizure prophylaxis in PML remain lacking [16]. In our case, the patient developed status epilepticus, emphasizing the severity of neurological involvement.

Her condition continued to deteriorate, with progressive decline in consciousness and recurrent seizures. Despite ART, co-trimoxazole, and supportive care, she developed aspiration pneumonia, which, combined with her critical neurological status and severe immunosuppression, ultimately led to her death.

This case underscores the need to consider HIV-associated opportunistic infections in the differential diagnosis of unexplained neurological symptoms. It also highlights the importance of a multidisciplinary approach in managing such complex presentations.

## Conclusions

This case highlights the importance of considering HIV and related infections, even in regions of reported low prevalence, such as the UK, in patients presenting with new neurological symptoms, particularly when the cause is unclear. Early testing for HIV and other opportunistic infections can help prevent delays in diagnosis and treatment. Management of these complex cases often requires a team-based approach involving multiple specialties. Early recognition of conditions such as PML and toxoplasmosis can improve patient care, although outcomes may remain poor in advanced disease.
